# Immune checkpoint inhibitor-induced dyshidrotic eczema following tremelimumab therapy for hepatocellular carcinoma

**DOI:** 10.1016/j.jdcr.2024.12.030

**Published:** 2025-01-15

**Authors:** Raphaella Lambert, Scott Stratman, Grace Rabinowitz, Hannah Verma, Jonas A. Adalsteinsson, George Niedt, Benjamin Ungar, Nicholas Gulati

**Affiliations:** Department of Dermatology, Icahn School of Medicine at Mount Sinai, New York, New York

**Keywords:** cutaneous immune-related adverse event, eczema, hepatocellular carcinoma, immune checkpoint inhibitor, tremelimumab

## Introduction

Tremelimumab is an anti-cytotoxic T-lymphocyte–associated antigen-4 (CTLA-4) monoclonal antibody within the category of antineoplastic therapeutics known as immune checkpoint inhibitors (ICIs).^1^ Tremelimumab is approved for the management of hepatocellular carcinoma and non-small cell lung cancer when used in conjunction with durvalumab, an ICI and programmed death ligand 1 monoclonal antibody.^1^ ICIs are used in the management of numerous solid and hematologic malignancies; however, such therapies are associated with cutaneous immune-related adverse events (cirAEs) including eczema, pruritus, psoriasis, vitiligo, bullous pemphigoid, and lichenoid eruptions.^2^^,^^3^ These reactions may vary in timing, presentation and severity, although they often require acute dermatologic evaluation.^2^^,^^3^

Eczematous reactions have been observed in patients treated with various ICIs, including pembrolizumab, ipilimumab, nivolumab, and durvalumab.^2^^,^^3^ Literature characterizing the cutaneous reactions induced by tremelimumab is limited.^4^^,^^5^ We report a case of dyshidrotic eczema induced by tremelimumab use.

## Case report

**Fig 1:**
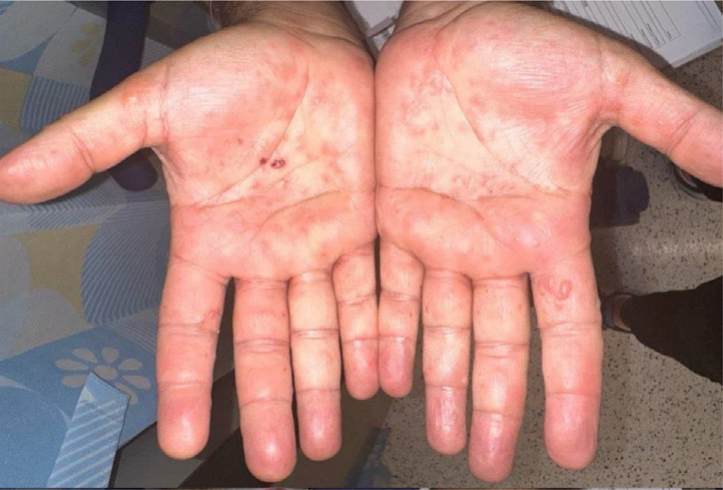
Vesicular palmar rash.

A 67-year-old man with a previous medical history of well-controlled HIV on bictegravir, emtricitabine, and tenofovir alafenamide, nonalcoholic steatohepatitis, and recurrent, metastatic hepatocellular carcinoma presented for evaluation of a pruritic, blistering rash involving the palms. The patient was diagnosed with hepatocellular carcinoma 4 years prior during routine nonalcoholic steatohepatitis screening and was treated with transarterial chemoembolization and radiofrequency ablation with good initial therapeutic response. Given subsequent progression of disease with metastasis to the left adrenal gland, he began immunotherapy with durvalumab 1500 mg every 4 weeks, with plan to add a single dose of tremelimumab 300 mg. The patient received his first durvalumab infusion without complication. Several days after his second durvalumab and first tremelimumab infusion, he experienced progressive symptoms of rash on the palms, believed to be secondary to tremelimumab infusion. Examination revealed clear, fluid-filled vesicles on the bilateral palms (Fig 1).

**Fig 2:**
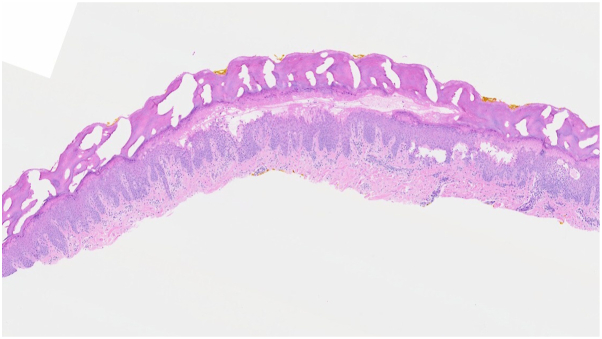
Diffusely acanthotic epidermis with intense spongiosis, intercellular edema, and vesicle formation, with underlying perivascular inflammation.

**Fig 3:**
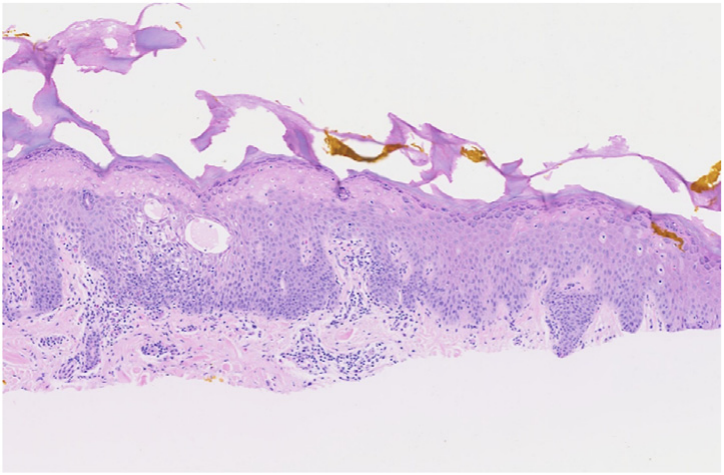
Intercellular edema, spongiotic vesicles, and scattered eosinophils.

Shave biopsy of a vesicle from the left second digit revealed spongiotic dermatitis with eosinophils. Microscopic evaluation was notable for superficial necrosis of the epidermis, epidermal acanthosis, intercellular edema, and a perivascular mononuclear infiltrate within the dermis (Figs 2 and 3).

Clinicopathologic correlation was consistent with dyshidrotic eczema; upon questioning, the patient denied any excessive hand washing or exposure to irritants. As such, irritant contact dermatitis was clinically excluded. Treatment with triamcinolone 0.1% topical ointment twice daily led to resolution of the rash within 2 weeks. The patient has continued durvalumab infusions without issue.

## Discussion

ICIs cause upregulation of the immune response, leading to cirAEs.^2^^,^^3^ CirAEs occur in up to 68% of patients treated with ICIs, with most reactions being pruritic and those that are non-specific in nature.^2^^,^^6^ Although less common, eczematous reactions in the setting of ICIs are most often observed as scaly macules and papules but can range from localized patches and plaques to nummular plaques and asteatotic eczema.^7^ Reports of dyshidrotic vesicles or morphologies are limited.^7^ Although clinical presentation of cirAEs can be nonspecific, most rashes are low grade (Common Terminology Criteria for Adverse Events Grade 1-2) and occur mainly on the trunk, with less frequent involvement of the extremities, digits, or face.^2^ Lesions typically appear after the first few ICI treatments, with rashes caused by anti-CTLA-4 agents often appearing sooner than those caused by anti-PD-1 agents (5 weeks with anti-PD-1 vs 3-4 weeks with anti-CTLA-4 antibodies).^2^ Eczematous reactions are histopathologically defined by spongiotic dermatitis with sparse to abundant eosinophils and perivascular T-lymphocyte infiltrate,^2^ consistent with the present case. Such eruptions are often self-limited, with good response to oral antihistamines and topical steroids in most patients. Cessation of ICI treatment is rarely required; some literature suggests that development of cirAEs may portend better prognostic outcome.^7^ However, clinical severity of cirAEs must be thoroughly evaluated, as systemic corticosteroids or biologic agents may be warranted for severe, persistent reactions. Further, clinicopathologic and laboratory evaluation may be necessary to rule out life-threatening cutaneous reactions including Stevens-Johnson syndrome, toxic epidermal necrolysis, or drug reaction with eosinophilia and systemic symptoms.^2^^,^^7^

Other cirAEs observed in the setting of programmed death ligand 1 inhibitor therapy, including durvalumab, have included vasculitis, dermatomyositis, lupus erythematosus-like eruptions, acneiform eruptions, lichenoid eruptions, psoriasiform eruptions, and bullous pemphigoid.^7^ Literature characterizing cirAEs resulting from tremelimumab therapy is limited. A single center, retrospective review of 17 patients treated with tremelimumab with resultant cirAEs observed pruritus (12/17), eczematous dermatitis (8/17), morbilliform rash (5/17), vitiligo (2/17), xerosis (3/17), acneiform rash (2/17), and psoriasiform dermatitis (1/17) among their cohort.^4^ None of the patients with eczematous dermatitis were noted to have a vesicular appearance consistent with dyshidrotic eczema.^4^ Six of the 17 patients were being treated with additional ICIs during the course of their tremelimumab therapy, including 4 concurrently receiving durvalumab.^4^ The average time to cirAE onset was 15 weeks, with an average of 54 weeks to clearance among patients with eczematous reactions.^4^ Only one patient required cessation of tremelimumab therapy given the severity of their rash.^4^

Identifying the causative agent behind cirAEs in the setting of multiple immunotherapies presents a diagnostic challenge.^4^ Given the resumption of this patient’s durvalumab treatment without continuation of rash, tremelimumab was favored as the trigger. Despite this, an important limitation of this case is the absence of a tremelimumab rechallenge; however, tremelimumab was intended as a single dose. The mechanisms underlying cirAE development secondary to CTLA-4 agents are incompletely understood, although research suggests some cirAEs may be related to the loss of T-cell tolerance.^4^^,^^8^ Such as other CTLA-4 agents including ipilimumab, tremelimumab induces T-cell proliferation to increase autoimmune tumor response. Tremelimumab is an IgG2 antibody and ipilimumab is an IgG1 antibody, with research suggesting that IgG2 antibodies result in less complement activation and less complement-dependent cytotoxicity as compared with IgG1 antibodies.^4^ Thus, IgG2 antibodies such as tremelimumab may lower the risk of cytokine-release syndrome, which should theoretically lead to fewer cirAEs, but may also decrease tremelimumab’s antineoplastic efficacy.^4^

With many cancer treatment paradigms favoring immunotherapies, dermatologists must be aware of the variety of cirAEs that may occur as well as their management. Literature on dyshidrotic eczematous reactions in the setting of immunotherapy is scarce. We highlight this case to add to the list of possible programmed death ligand 1 and CTLA-4 inhibitor cirAEs, and to improve both provider recognition of these reactions as well as patient outcomes.

## Conflicts of interest

None disclosed.
